# Multistationary and Oscillatory Modes of Free Radicals Generation by the Mitochondrial Respiratory Chain Revealed by a Bifurcation Analysis

**DOI:** 10.1371/journal.pcbi.1002700

**Published:** 2012-09-20

**Authors:** Vitaly A. Selivanov, Marta Cascante, Mark Friedman, Mark F. Schumaker, Massimo Trucco, Tatyana V. Votyakova

**Affiliations:** 1 Department of Biochemistry and Molecular Biology, Faculty of Biology, Universitat de Barcelona, Barcelona, Spain; 2 Institute of Biomedicine of Universitat de Barcelona (IBUB) and CSIC-Associated Unit, Barcelona, Spain; 3 A.N.Belozersky Institute of Physico-Chemical Biology, Moscow State University, Moscow, Russia; 4 Mathematical Sciences Department, University of Alabama Huntsville, Huntsville, Alabama, United States of America; 5 Department of Mathematics, Washington State University, Pullman, Washington, United States of America; 6 Department of Pediatrics, The University of Pittsburgh School of Medicine and The Children's Hospital of Pittsburgh, Pittsburgh, Pennsylvania, United States of America; University of California San Diego, United States of America

## Abstract

The mitochondrial electron transport chain transforms energy satisfying cellular demand and generates reactive oxygen species (ROS) that act as metabolic signals or destructive factors. Therefore, knowledge of the possible modes and bifurcations of electron transport that affect ROS signaling provides insight into the interrelationship of mitochondrial respiration with cellular metabolism. Here, a bifurcation analysis of a sequence of the electron transport chain models of increasing complexity was used to analyze the contribution of individual components to the modes of respiratory chain behavior. Our algorithm constructed models as large systems of ordinary differential equations describing the time evolution of the distribution of redox states of the respiratory complexes. The most complete model of the respiratory chain and linked metabolic reactions predicted that condensed mitochondria produce more ROS at low succinate concentration and less ROS at high succinate levels than swelled mitochondria. This prediction was validated by measuring ROS production under various swelling conditions. A numerical bifurcation analysis revealed qualitatively different types of multistationary behavior and sustained oscillations in the parameter space near a region that was previously found to describe the behavior of isolated mitochondria. The oscillations in transmembrane potential and ROS generation, observed in living cells were reproduced in the model that includes interaction of respiratory complexes with the reactions of TCA cycle. Whereas multistationarity is an internal characteristic of the respiratory chain, the functional link of respiration with central metabolism creates oscillations, which can be understood as a means of auto-regulation of cell metabolism.

## Author Summary

The mitochondrial respiratory chain shows a variety of modes of behavior. In living cells, flashes of ROS production and oscillations accompanied by a decrease of transmembrane potential can be registered. The mechanisms of such complex behavior are difficult to rationalize without a mathematical formalization of mitochondrial respiration. Our most complete model of mitochondrial respiration accounts for the details of electron transport, reproducing the observed types of behavior, which includes the existence of multiple steady states and periodic oscillations. This most detailed model contains hundreds of differential equations, and such complexity makes it difficult to grasp the main determinants of its behavior. Therefore the full model was reduced to a simplified description of complex III only, and numerical bifurcation analysis was used to study its behavior. Then the evolution of its behavior was traced in a sequence of models with increasing complexity leading back to the full model. This analysis revealed the mechanism of switching between the modes of behavior and the conditions for persistence in a given state, which defines ATP production, ROS signaling and destructive effects. This is important for understanding the biochemical basics of many systemic diseases.

## Introduction

**Figure 1:**
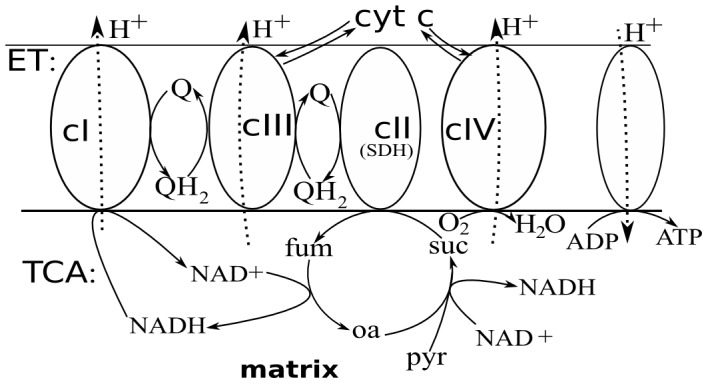
Scheme of electron transport chain (ET) and linked reactions of TCA cycle. Respiratory complex I (cI) oxidizes NADH and reduces ubiquinone (Q), translocating H^+^. Complex II (cII) also reduces Q, oxidizing succinate (suc) to fumarate (fum). The net effect of complex III (cIII) is to oxidize ubiquinol (QH_2_), reducing cytochrome c and translocating H^+^. Complex IV (cIV) oxidizes cytochrome c, reducing molecular oxygen to H_2_O and translocating H^+^. The H^+^ gradient thus created is used for ATP synthesis. NADH is consumed by complex I and is produced in the several reactions of TCA cycle leading from pyruvate (pyr) to suscinate, and from fumarate to oxaloacetate (oa).

The electron transport chain links the central carbohydrate energy metabolism with ATP synthesis (see Fig. 1). It transforms the free energy released by the oxidation of NADH and succinate into a form of transmembrane electrochemical potential (ΔΨ), which is used for ATP synthesis [1]. Reactive oxygen species (ROS) are byproducts of electron transport [2]. They play the roles of both metabolic signals and destructive agents [3]–[8]. These key roles of electron transport in cellular metabolism motivate the great interest in understanding the details of the dynamics of this process. Electron flow through the chain of carriers is controlled by levels of substrates (NADH, succinate), levels of tricarboxylic acid (TCA) cycle intermediates, the rate of ATP consumption, oxygen availability, etc [9]. However, many interesting dynamical properties of the electron transport and linked ROS production are determined by the intrinsic properties of the electron transport chain, such as the structural and functional links between carriers (topology of the system) and values of parameters, e.g. reaction rate constants. Such intrinsic properties can determine physiologically important modes of respiratory chain operation and how transitions between these modes occur. This relationship between intrinsic properties and dynamics can be understood by analyzing a detailed model of electron transport. We have reported elsewhere that the Q-cycle mechanism of electron transport in respiratory complex III exhibits bistability [10], i.e. two stable steady states may exist under the same microenvironmental conditions (corresponding to two stable steady state solutions of a system of ordinary differential equations (ODEs) at the same parameter values). The importance of bistability resides, in particular, in the fact that it can be a main determinant of the destructive effects of hypoxia/reoxygenation [3], [11]. Bistability also occurs in a model of the whole respiratory chain that agrees quantitatively with the measured forward and backwards fluxes through the respiratory chain [12].

Current experimental techniques make it possible to monitor the behavior of a single mitochondrion in living cells [13]. This has provided evidence that mitochondria can operate in numerous qualitatively distinct modes. They can persist in a steady state characterized by a high value of ΔΨ and a low rate of ROS production, can switch to another steady state characterized by a low value of ΔΨ and a high rate of ROS production, and can also enter into a mode of sustained oscillations [13]–[15]. The two qualitatively different steady states can be associated with the normal physiological state and a pathological one that can be approached after hypoxia/reoxygenation. The oscillatory behavior is probably very important for intracellular signaling, as it was found for Ca^2+^ signaling [16]. An application of a systematic method that reveals qualitatively different modes of model behavior and corresponding regions of relevant problem parameters, i.e. a bifurcation analysis of a mathematical model describing mitochondrial electron transport, would give insight into these physiologically important modes of mitochondrial functioning and the mechanism of switching between modes. The objective of the present study is to advance in this direction.

**Figure 2:**
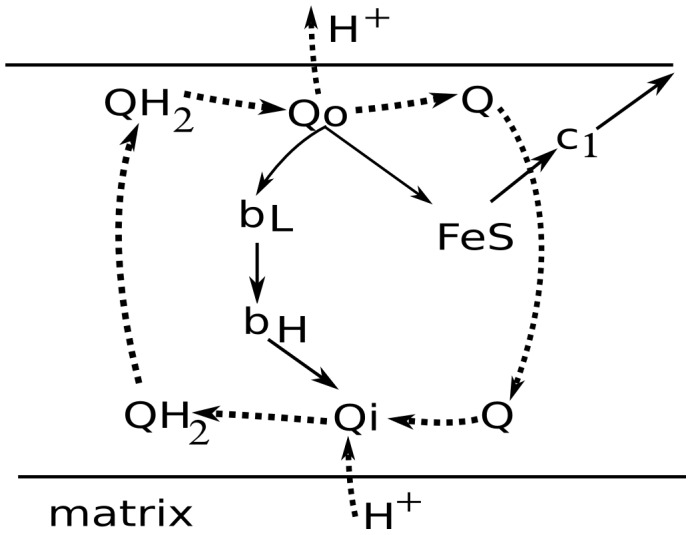
The implemented scheme of electron and proton transport catalyzed by complex III. The redox centers included in the model of complex III: Qo is the site of quinol (QH_2_) oxidation and ubiquinone (Q) production, b_L_ and b_H_ are the two hemes of cytochrome b, FeS is the redox center of the Rieske protein, and c_1_ is the cytochrome c_1_. Solid arrows indicate the pathways of electron flow. Dotted arrows indicate the binding/dissociation of Q/QH_2_ and H^+^. The arrows leading out from c_1_ indicate electron flow to oxygen through the implied other components of the respiratory chain.

This paper presents a bifurcation analysis of four increasingly complicated models. The first two of these describe only complex III (Fig. 2), in forms simplified compared with those previously presented [10]. The last two include elements of the respiratory chain, shown in Fig. 1, that were previously modeled in [12]. For respiratory complex III, the models assume that the core of the complex contains four redox sites: cytochrome b with its two hemes, b_H_ and b_L_, cytochrome c_1_, and the Rieske protein containing iron-sulfur center (b_H_-b_L_-c_1_-FeS). In addition, the core can bind a two-electron carrier ubiquinone either in the matrix (Qi-Qi-) or cytosolic (-Qo-Qo) side of the inner mitochondrial membrane. Symbols are repeated to represent the two electrons. This gives four different configurations of complex III: (b_H_-b_L_-c_1_-FeS, b_H_-b_L_-c_1_-FeS-Qo-Qo, Qi-Qi-b_H_-b_L_-c_1_-FeS, Qi-Qi-b_H_-b_L_-c_1_-FeS-Qo-Qo). The models take into account that respiratory complexes constituting the respiratory chain consist of a number of fixed in space electron carriers that can be either reduced (red) or oxidized (ox). A possible state of a complex is defined by a combination of redox states of individual carriers constituting it. The model variables correspond to these states. The repeated symbols each correspond to three possible states: either with two valence electrons and two corresponding protons (ubiquinol), one valence electron (semiquinone), or no valence electrons (ubiquinol). Thus the configuration Qi-Qi-b_H_-b_L_-c_1_-FeS-Qo-Qo has 144 possible states, each corresponding to a variable in the model.

These large numbers of variables make it difficult even to write down explicitly the corresponding systems of ordinary differential equations (ODEs). Therefore an algorithm is designed and implemented to automatically compute the values of the right hand sides of the ODE system at each step of a numerical solution of a corresponding initial value problem (IVP), based on the rules formulated in accordance with the reaction mechanism [10]. Two complimentary techniques are combined for numerical bifurcation analysis to systematically search for various types of model behavior and for intervals of parameter values corresponding to multiple steady state solutions or oscillatory solutions: 1) using IVP solvers to solve IVPs for our ODE systems, and 2) using the numerical bifurcation analysis software CL-MATCONTL [17], [18] (Text S1) to study the corresponding large equilibrium systems.

## Results

### Complex III model

**Figure 3:**
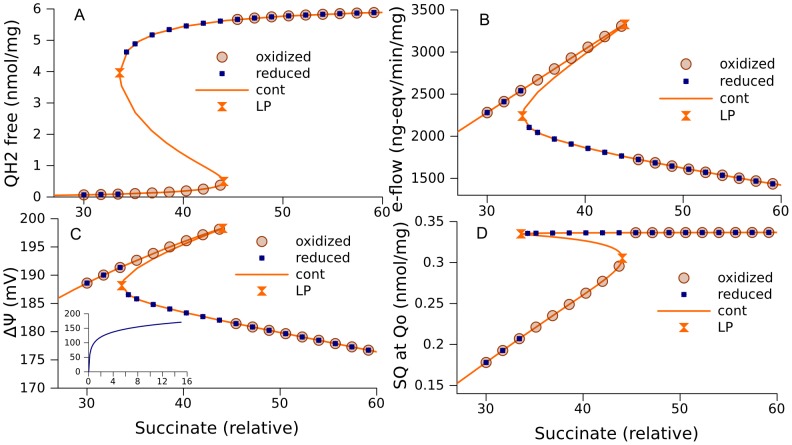
Multiple steady state solutions corresponding to the simplified model of complex III (model 145). A, steady states for ubiquinol concentrations as a function of relative succinate concentration (from eq (1) in Methods, k = 80); B, net electron flow through the complex III; C, ΔΨ; the inset also shows ΔΨ in the interval (0, 15) of relative succinate concentrations; D, semiquinone (SQ) concentrations formed at Qo site. The orange curves are obtained by continuation of steady state (cont) using CL_MATCONTL. The points labeled “reduced” and “oxidized” are obtained as the steady states approached by the solution of an initial value problem starting from a point where ubiquinone is almost completely in the reduced (QH_2_) or oxidized (Q) form of the electron carrier, respectively. Symbols “LP” designate limit points. The values of other parameters are shown in Table 1 (column “145”).

A numerical bifurcation analysis of the respiratory chain was first conducted for a model of complex III simplified to 145 equations (referred to further as model 145) as described in Methods, with basic set of parameters listed in Table 1. This model accounts for only one configuration of complex III, namely the core (consisting of cytochrome b with its two hemes b_H_ and b_L_, cytochrome c_1_, and the Rieske iron-sulfur (FeS) center) together with ubiquinone molecules bound at both inner (i) and outer (o) sites. This configuration is denoted as Qi-Qi-b_H_-b_L_-c_1_-FeS-Qo-Qo. Binding/dissociation of quinones in this model is accounted for by the replacement of reduced bound molecules at Qi by oxidized bound molecules and oxidized bound molecules at Qo by reduced bound molecules (see Methods). Numerical continuation of steady state solutions, as a function of succinate concentration, (proportional to Vm_SDH_, eq.(1)), performed with CL_MATCONTL (as described in Methods), revealed an interval of parameter values with multiple steady state solutions (Fig. 3, orange curves) enclosed between two limit points (LP) indicating the points of fold bifurcations. This interval of the parameter values for which multiple steady state solutions exist, corresponds to two curve segments of stable steady states and one curve segment of unstable steady states in between.

**Table 1:** Rate constants of elementary reactions taking place in complex III.

Model:	145	257	267/272
kqo_FS	348565	316575	316897
kFS_qo	11515	43575	43852
kFS_c1	510160	509775	510162
kc1_FS	150780	137025	137082
kqo_bl	69090	68775	69099
kbl_qo	3220	1091	3225
kbl_bh	87675	87675	87688
kbh_bl	1155	539	1156
Kbh-qi1	99540	891608	90521
kqi1_bh	29995	4056	30015
kbh_qi2	298200	297675	298139
kqi2_bh	27090	32498	27093
kc1c	84525	112538	257
klk	1400	1417	1417
kqhob		172200	2793
kqhod		2326	2585
kqib		12600	12682
kqid		237	180
kqod		24554	3525
kqob		4	740
kqhid		3768	3768
kqhib		17770	6912

Units for the rate constants of monomolecular reactions of electron transition inside the complex and reactions of dissociation are s^−1^. For bimolecular reactions of ubiquinone/ubiquinol binding units are (nmol/mg)^−1^s^−1^. The reactions simulated and abbreviations used in the names of constants are explained in Methods, part “Complex III model”.

### Mechanism of bifurcations

The method of numerical bifurcation analysis that we used allowed us to accurately and rigorously determine the bifurcation behavior of the whole system, without any simplifications. At the same time, the main process underlying the bifurcation behavior can, in some cases, be heuristically identified by reducing a system by taking into account different time scales. Such a reduction of Model 145 (see Text S2) points to binding/dissociation of ubiquinone coupled with its reduction/oxidation as the main process responsible for the multistationarity of complex III (Fig. S1 in Text S2).

A gradual increase of succinate from low concentrations towards the interval of multistationarity leads to the lowest branch of steady states for quinol (QH_2_) as shown in Fig. 3A. Under such conditions complex III functions to give high electron flow and high ÄØ (upper branches in Figs. 3B and 3C). It should be noted that, although the concentrations of QH_2_ that constitute the lower branch of steady states in Fig. 3A are low, they nevertheless are sufficient to maintain high levels of ΔΨ. The inset in Fig. 3C shows that ΔΨ drops to 0 as the concentration of succinate decreases to 0. This drop is a consequence of QH_2_ deficiency. A decrease of succinate concentration from 15 to 0 corresponds to the almost linear decrease of QH_2_ levels from 0.02 to 0 nmol/mg prot. The above described “active” state, that provides highest electron flow, is characterized by low levels of semiquinone (SQ) at the quinol oxidase site (Qo) (Fig. 3D).

If succinate concentration surpasses the right limit point on Fig. 3A, the rate of ubiquinone reduction by succinate dehydrogenase outstrips the maximal capacity of its oxidation by complex III, therefore Q is almost completely converted into QH_2_, as Fig. 3A shows. This is the biochemical mechanism of the bifurcation. The lack of an electron acceptor at Qi site results in a decrease of electron flow through the Q-cycle, a decrease of ΔΨ, and an increase in levels of SQ at Qo. If then succinate concentration decreases, the blocked electron transport cannot produce a sufficient amount of acceptors Q to activate electron flow. When the system is in such a blocked state, even decreased succinate dehydrogenase activity is sufficient to maintain low levels of Q and keep the system blocked. If initially the electron carriers are oxidized, the solution of an initial value problem for the ODE system approaches an “active” steady state, and if initially the carriers are reduced, the solution approaches a “blocked” steady state.

Model 145 has 13 parameters. The number of parameters is much less than the number of equations because parameters characterize the types of electron transport reaction, which is a much smaller number than the number of combinations of redox states of carriers (the number of equations). Different states participate in reactions of the same type with the same parameters, but they cannot be combined (the system cannot be reduced without additional simplifications) because the whole set of reactions for each state (variable) is different.

Fig. 3 shows curve segments of multiple steady state solutions corresponding to an interval of values of one parameter. The shape and size of these curve segments depends on the values of other parameters. The width of this interval may be smaller, or the interval may even disappear. Fig. 3 shows an interval of relative succinate concentrations corresponding to multiple steady states, obtained using an algorithm (described in Methods) that scans all the parameters with the objective of finding as large interval as possible.

**Figure 4:**
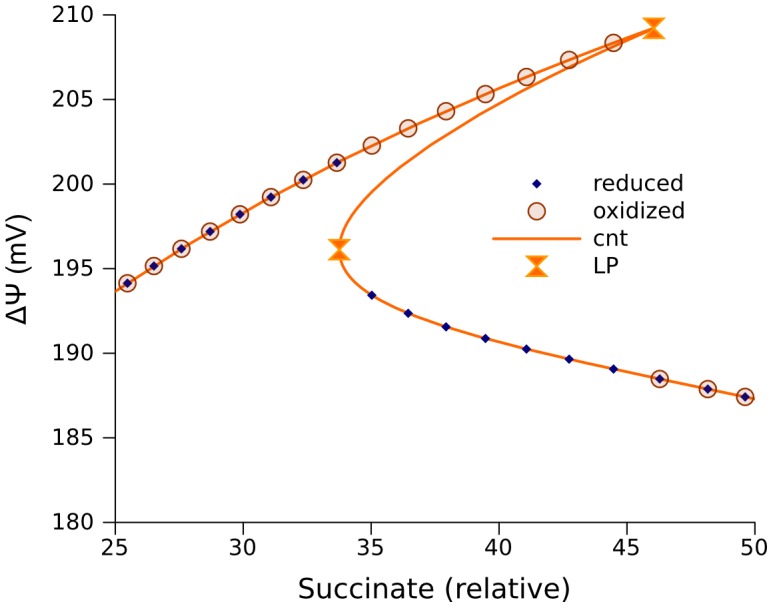
Multiple steady state solutions for the model of complex III consisting of 257 ODEs. The solutions for ÄØ are given as a function of succinate concentration defining Vm_SDH_ (eq (1)). The values of other parameters are shown in Table 1 (column “257”).

Including in our model all of the four configurations of complex III and an explicit description of quinone binding/dissociation increases the number of equations to 257 (see Methods). This more detailed model also has a region of multiple steady state solutions. Application of the same algorithm maximizing the region of relative succinate concentration characterized by multiple steady states resulted in the interval that is larger than the one in the case of model 145, as is shown for ΔΨ in Fig. 4. However, the qualitative behavior of the two models remains similar. Evidently, model 145 faithfully accounts for the main properties of complex III determined by the Q-cycle mechanism.

### Including complex I

**Figure 5:**
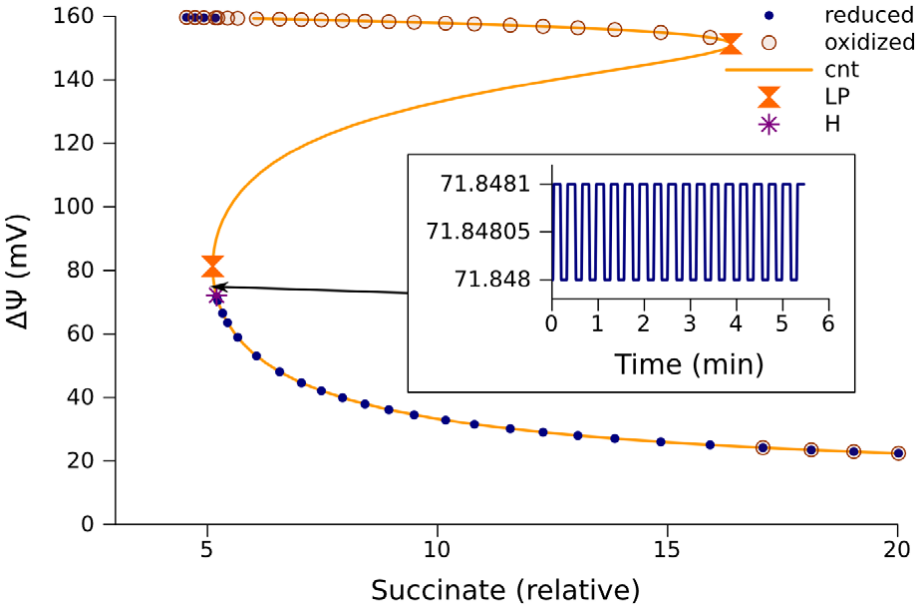
Multiple steady states for the integrated models of complex I and III (model 267). The solutions for ΔΨ are given as a function of succinate concentration defining Vm_SDH_ (eq (1)). The rate constant of electron transport from cytochrome c1 to cytochrome c (k_c1c_) was 735 s^−1^. The values of other parameters, which correspond to the best fit to measured dynamics of NADH and oxygen consumption under states 4 and 3 of mitochondrial respiration [12], are shown in Table 1 (column “267/272”) and Table 2. The point “H” designates a Hopf bifurcation, and the inset shows the time course of ΔΨ (mV) in the neighborhood of this point.

Model 267 is obtained by adding to model 257 equations that account for reactions taking place in complex I (described in Methods). This extended model contains almost all of the essential components of the respiratory chain model that we used for the analysis of experimental data [12]. Using it enables us to start the numerical bifurcation analysis for a “real” set of parameter values (Table 2) that reproduces the measured dynamics of NADH reduction in the presence and absence of rotenone, and the maximal and state 4 respiration rates, when mitochondria are fueled by succinate or pyruvate/malate [12]. Numerical continuation of steady state solutions for ΔΨ as a function of succinate concentration uncovers the existence of an interval of parameter values with multiple solutions, in the form of an S-shaped curve, enclosed between two limit points (Fig. 5). There is also a Hopf bifurcation point in the vicinity of one limit point. The sustained oscillation, which can be simulated in the parameter interval between the left limit point and Hopf bifurcation, has very small amplitude (inset in Fig. 5). The mechanism of the fold bifurcations in this case is similar to that discussed for the simplest model. Similar to the case presented in Fig. 3, the stable steady states with the lowest values of ÄØ have the highest levels of SQ radicals at the Qo site; this may be a physical basis for high ROS production rates.

**Table 2:** Rate constants of elementary reactions taking place in complex I.

Parameter	Value
kfmn	368553
krfmn	508076
kf_n2	99177
kn2_f	4669
kn2_q1	500906
kq1_n2	51112
kqpqn	31657
krqnqp	116
kqhdis	213647
kqhbnd	20398
kqbnd	201029
kqdis	2829
kn2_q2	146954
kq2_n2	2159

Units for the rate constants of monomolecular reactions of electron transition inside the complex and reactions of dissociation are s^−1^. For bimolecular reactions of ubiquinone/ubiquinol binding units are (nmol/mg)^−1^s^−1^. The reactions simulated and abbreviations used in the names of constants are explained in Methods, part “Including complex I”.

### Expected variations of bifurcation behavior

**Figure 6:**
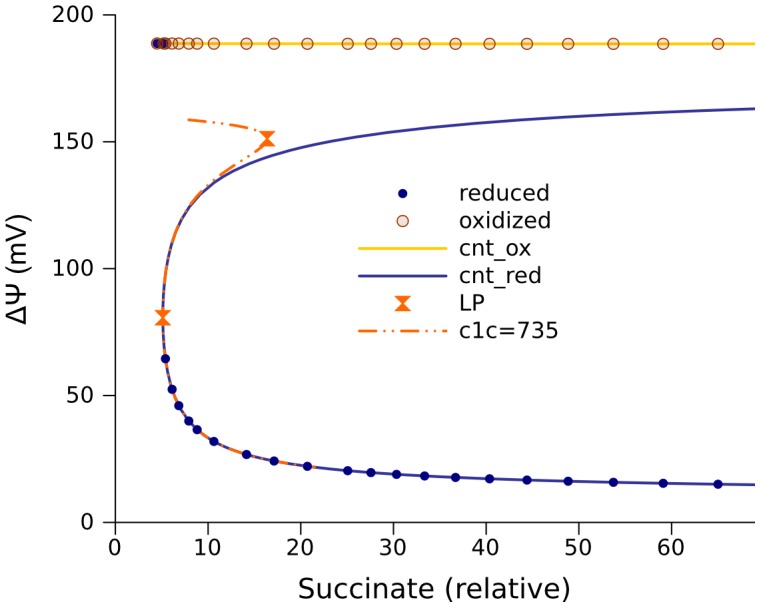
Infinite interval of multiple steady states for the model 267. The solutions for ΔΨ are given as a function of succinate concentration (defining Vm_SDH_ in equation (3)). The rate constant of electron transport from cytochrome c1 further to cytochrome c (k_c1c_) was 4200 s^−1^. The values of other parameters, the same as for Fig. 5, are shown in Tables 1 and 2. The dash-dot-dot curves are the same as orange curve in Fig. 5.

The measurements that were used to find the given set of parameters were performed in a suspension of isolated mitochondria. The rate of electron transport from cytochrome c1 to cytochrome c is the parameter most affected by the procedure of isolation, since it depends on the structure of intermembrane space, which is significantly changed after the isolation. In intact mitochondria the value of this parameter is expected to be higher than in isolated mitochondria. Increasing its value by less than an order of magnitude increases the interval of multiple steady state solutions to infinity (Fig. 6). Starting from an initially oxidized state, the system approaches a steady state, which, with numerical continuation with the substrate concentration as a parameter, results in the upper branch in Fig. 6. This branch does not contain any bifurcation points. However, at a high substrate concentration, and starting from a reduced state, the system approaches another steady state located on a different branch marked blue in Fig. 6. The lower segment of this branch is stable. A decrease of the substrate supply parameter ultimately leads to a limit point, and the upper segment of unstable steady states starts at this point. Note, this branch of unstable steady states is not connected to the upper branch of stable steady states. Similar to the cases analyzed above, the steady states corresponding to low ΔΨ values (lowest blue branch) are characterized by practically complete reduction of the free ubiquinone pool and maximal levels of free radicals SQ at the Qo site. Similarly to the cases analyzed above, the steady states corresponding to high ΔΨ (yellow branch) are accompanied by an oxidized free ubiquinone pool and low levels of SQ at the Qo site.

The change of a single parameter that characterizes interaction between cytochromes c1 and c (k_c1c_) gives a qualitatively different behavior of model 267, as seen in Fig. 6 from the comparison between the blue curve and the orange curve, which is redrawn from Fig. 5. Such a difference in behavior can be induced by swelling/shrinking of mitochondria, as was mentioned above, but also hypoxia/reoxygenation can induce a similar change of parameters and, thus, similar effects. Hypoxia in our model can be simulated as a k_c1c_ decrease.

### Application to hypoxia/reoxygenation

Assume that before hypoxia the system effectively functions at some point on the yellow curve (Fig. 6). Suppose the change of k_c1c_ induced by hypoxia transforms the properties of the system so that the orange curve becomes the continuum of its steady states. If before hypoxia the functional steady state was to the right of the rightmost limit point in orange curve, then after hypoxia the system evolves until it reaches a steady state in the lower segment of orange curve (coinciding with the lower segment of blue curve). If then the system is re-oxygenated, and the blue and yellow curves again become the continuum of steady states, it remains in the same state now located in the low segment of blue curve. Thus, hypoxia and re-oxygenation change the state of the system. Before hypoxia it generated high ΔΨ (yellow curve in Fig. 6) and slowly produces ROS, whereas after re-oxygenation it stays in a state characterized by low ΔΨ (blue curve with points in Fig. 6) and rapidly produces ROS.

### Including the TCA cycle

**Figure 7:**
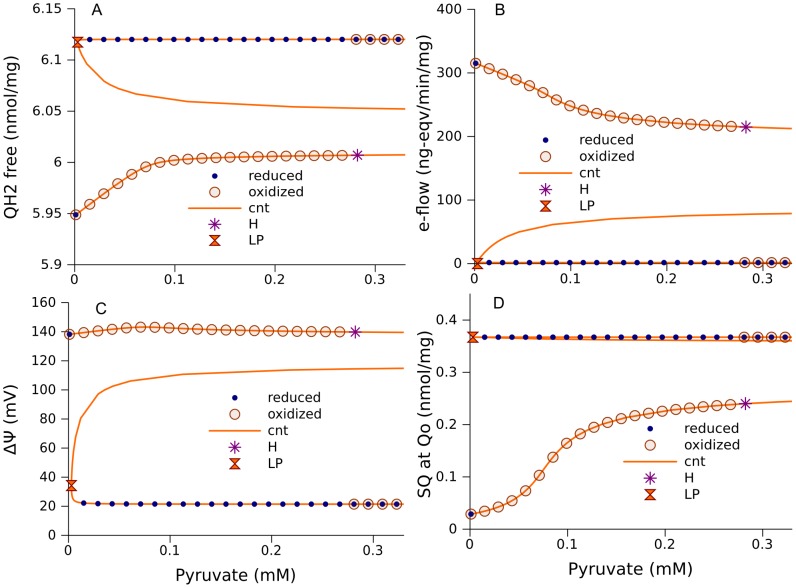
Multiple steady states at various pyruvate concentrations. The model 267 was extended to 272 equations by including the TCA cycle as described in Methods. The values of parameters are shown in Tables 1–3. They are the same as used elsewhere to fit the experimental dynamics of NADH and respiration rate [12]. A, steady states for ubiquinol concentrations as a function of relative pyruvate concentration; B, net electron flow through the complex III; C, ΔΨ; D, semiquinone (SQ) concentrations formed at Qo site. The orange curves are obtained by continuation of steady state (cnt) using CL_MATCONTL. The points indicated as “reduced” and “oxidized” are obtained as the steady states approached by the solution of an initial value problem starting from initially reduced or oxidized states of electron carriers respectively. Symbols “LP” designate limit points, “H” – Hopf bifurcation points.

Further extension of the ODE model to 272 equations, as described in Methods, by including the reactions of the TCA cycle with the parameters listed in Table 3, allowed us to study the interaction of the respiratory chain with central carbohydrate metabolism. In the extension, pyruvate is accounted for as a substrate for the TCA cycle, which provides succinate for complex II and NADH for complex I. Using parameter values verified by fitting the measured dynamics of NADH and respiration rates [12], this model predicts the existence of wide range of pyruvate concentrations with two stable steady state segments (Fig. 7), as well as those described above with respect to succinate.

**Table 3:** Parameters of TCA cycle reactions and substrate transport.

Parameter	Value	Units
kpyr	52	s^−1^
kCS	2109	mM^−1^ s^−1^
kTCA	876	mM^−1^ s^−1^
VmSDH	171	mM^−1^ s^−1^
kMDH	452	mM^−1^ s^−1^
ksuc	376	s^−1^
kME	0.000231	mM^−1^ s^−1^
ksm	7	mM^−1^ s^−1^
Kmsuc	0.5	mM
Kmq	0.5	nmol/mg

**Figure 8:**
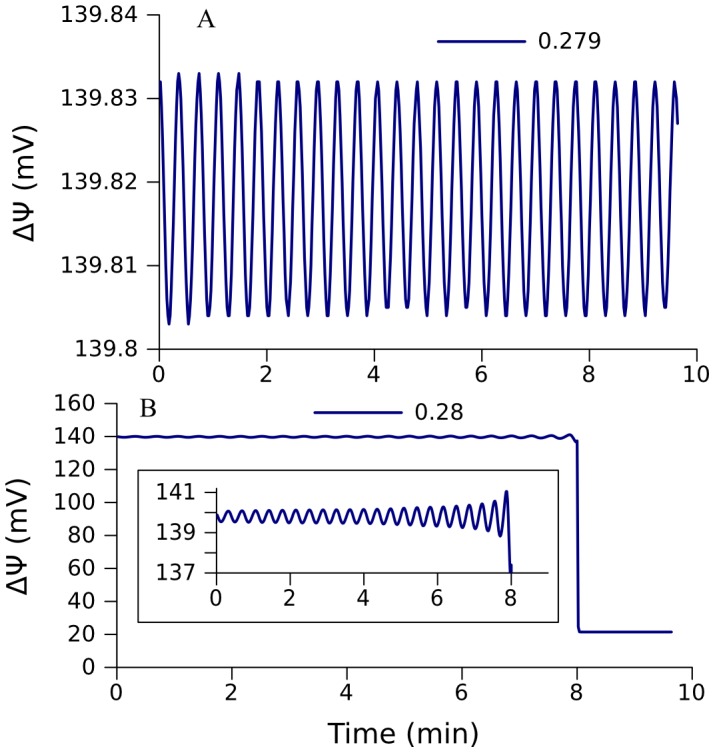
Dynamic behavior of model 272 in the neighborhood of the point of Hopf bifurcation indicated in Figure 7. Small-amplitude oscillations monitored by ΔΨ are stable at external pyruvate concentrations 0.279 mM (A), but the increase of amplitude induced by increase of pyruvate concentration to 0.28 mM results in the switch to another branch of steady states (B).The inset in B zooms the interval of oscillations of ΔΨ (ordinates, ΔΨ (mV); abscissa, Time (min)).

Similarly to the cases considered above, the redox state of free ubiquinone pool determines the dynamics of the system. If the free ubiquinone pool (Fig. 7A) is not completely reduced, the electron flow through the respiratory chain (Fig. 7B) and ÄØ (Fig. 7C) is high, and the level of SQ at the Qo site (Fig. 7D), determining the ROS production rate, is low. On the other hand, if ubiquinone is practically completely reduced, the electron flow through the respiratory chain and ÄØ is low, and the level of SQ at the Qo site is high. However, the bifurcations which determine a switch between the two steady state branches are different from those considered above. Specifically, when the system is in an oxidized state, the increase of the pyruvate concentration leads to a Hopf bifurcation. Oscillations in a neighborhood of this bifurcation point have insignificantly small amplitude (see Fig. 8A). An increase of the control parameter makes the amplitude greater (inset in Fig. 8B), but the trajectory comes to the zone of attraction of the lower segment of stable steady states (Fig. 7C) and approaches one of these states (Fig. 8B). As the pyruvate concentration decreases, the system stays in this “reduced” stable curve segment until it reaches the limit point at ∼0.003 mM, where a curve segment of unstable steady states starts.

### High-amplitude oscillations

**Figure 9:**
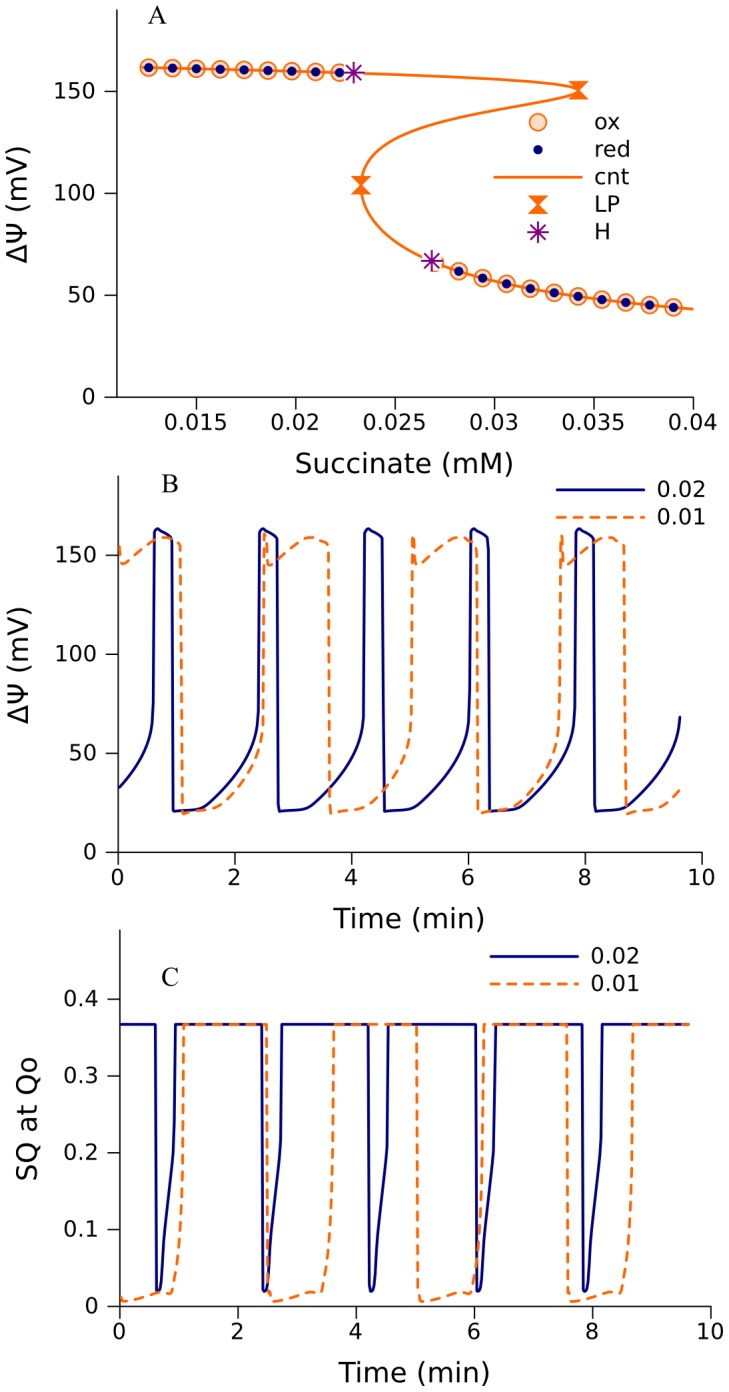
Sustained oscillations of levels of ÄØ and SQ bound at Qo site in model 272. A, bifurcation diagram in the space of succinate concentration as a parameter. Orange line indicates steady states obtained by numerical continuation using CL-MATCONTL (cnt), and circles mark the branches of stable steady states accessible from initially oxidized (ox) or reduced (red) states. Two Hopf bifurcation points (H) restrict the branches of stable steady states, rendering unstable the segments between the Hopf and limit points (LP) in the upper and lower branches. These two Hopf points restrict the interval of sustained oscillations exemplified for ΔΨ (panel B) and the levels of SQ bound at Qo site (panel C) at 0.024 mM of succinate. The numbers indicate pyruvate concentrations. The rate constant of electron transport from cytochrome c1 to cytochrome c (k_c1c_) was 782 s^−1^. Vm_SDH_ (equation (5)) was 1714 nmol/min/mg. The values of other parameters used are shown in Tables 1–3.

An increase of the cytochrome c1 to cytochrome c electron transition rate (k_c1c_) to 782 s^−1^ and an increase in Vm_SDH_ (equation (5)) from 171 (Table 3) to 1714 nmol/s/mg changes the bifurcation behavior as is shown in Fig. 9A. Although the bifurcation diagram here is also a basic S-shaped curve producing multi-stationarity, the entire segment of steady states between the two Hopf bifurcation points is unstable. Stable oscillations of high amplitude appeared between these Hopf bifurcation points (Fig. 9B and 9C). ÄØ oscillates between 160 and 20 mV; such changes can be measured and, probably, this mechanism underlies the observed behavior [19]. As Figs. 9B and 9C show, ÄØ and the level of SQ at the Qo site (defining the rate of ROS production) oscillate in counter-phase. This also corresponds to the behavior monitored in intact mitochondria [14]. Variation of parameters can significantly change the durations of phases of low or high potential (high or low ROS production rate, respectively). This could be a basis of the ROS signaling [20].

### Mechanism of oscillations

The mechanism of oscillations arises from an interaction of the ubiquinone reduction/oxidation with the TCA cycle. The switch from the “oxidized” curve segment of steady states to the “reduced” one is accompanied by a decrease of the electron flow, and, as a consequence, an increase of the NADH levels (decrease of NAD^+^). Since the conversion of pyruvate in the TCA cycle requires NAD^+^, the production of succinate slows down. The high levels of NADH are maintained for some time due to reverse electron transport through complex I reducing NAD^+^. A decrease of the substrate production in the TCA cycle and reverse electron transport result in an accumulation of a sufficient amount of the electron acceptor ubiquinone that activates electron transport, which results in switching back to the curve segment of the “oxidized” steady states, and then the cycle repeats again.

### Experiment and its simulation

**Figure 10:**
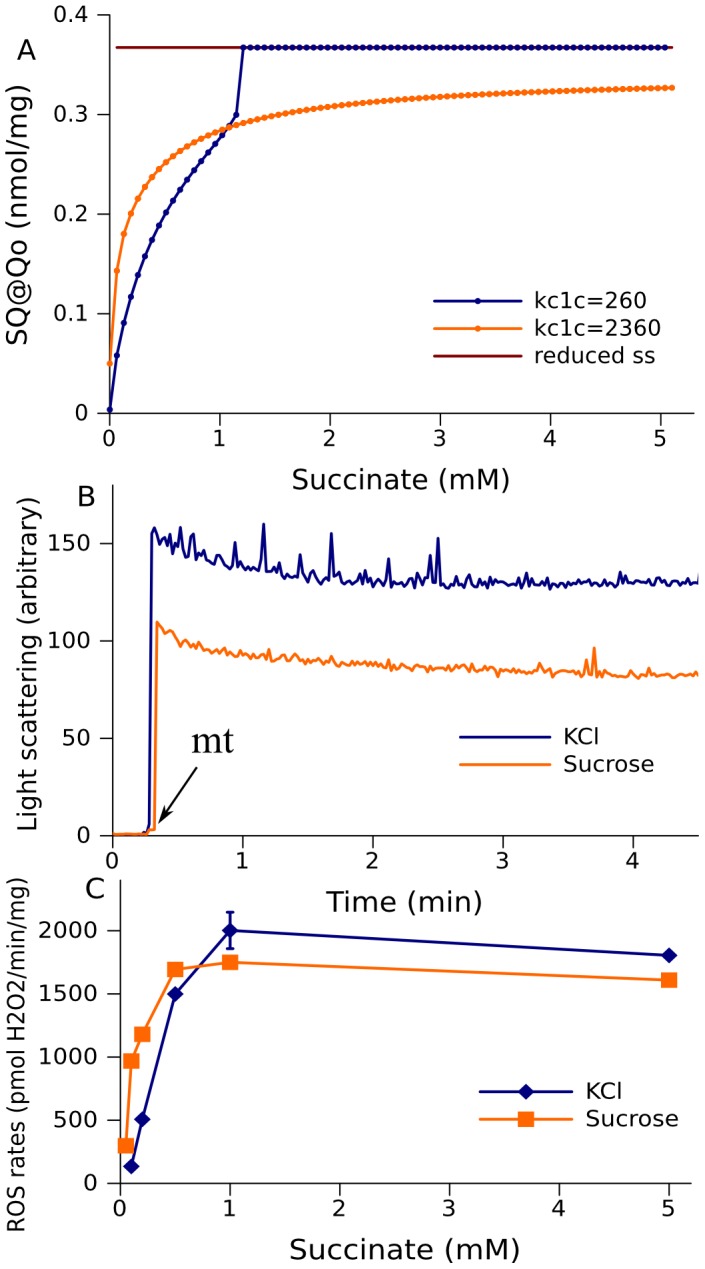
Compatibility between the calculated steady state levels of free radicals and measured ROS production rates. A, the levels of SQ at Qo site for model 272 are computed as a function of succinate concentrations with various rate constant of cytochrome c1 oxidation (k_c1c_). The branches of steady states reachable from initially oxidized states are different for the two cases shown (indicated with blue and orange curves), and the branches of steady states reachable from initially reduced states coincide (brown line). B, measured light scattering of mitochondrial suspension in KCl or sucrose media as described in Methods. C, measured ROS production rates by mitochondrial suspension in KCl or sucrose media as described in Methods.

The change from multi-stationarity shown in Fig. 7 to an oscillatory behavior shown in Fig. 9 is, in part, the result of a change of the rate constant k_c1c_, which accounts for interaction between cytochromes c_1_ and c. This rate constant depends on the volume of the intermembrane space, where the interaction takes place. The intermembrane volume can be controlled experimentally in a suspension of isolated mitochondria, and the correspondence of the model predictions and the measured ROS production rates under variations of the intermembrane space can be experimentally verified. The change of the ROS production rate (characterized by the level of SQ at the Qo site), predicted for an “oxidized” state of model 272 with an increase of succinate concentration, is shown in Fig. 10A. The shape of the lower curve segment of steady state concentrations of SQ bound at the Qo site depends on parameter k_c1c_. Fig. 10A shows a superposition of curve segments of stable steady states obtained at two different values of k_c1c_. At a low value of this parameter (∼260 s^−1^ as shown in Table 1), increasing the succinate concentration above 1 mM takes the system past the Hopf bifurcation point (similar to that shown in Fig. 7D), and it switches to the upper curve segment of SQ stable steady state concentrations (blue curve). Increasing k_c1c_ shifts this Hopf bifurcation point to infinity, so that the upper branch of stable steady states, although it still exists, becomes inaccessible from the lower branch in the space of succinate concentrations (orange curve).

In Fig. 10A, the lower branch of steady states obtained at a higher value of k_c1c_ crosses the lower branch obtained at a lower value of k_c1c_. At low succinate concentrations, the levels of SQ at the Qo site are higher when k_c1c_ is high. At high succinate concentrations, this relationship is reversed. We have confirmed this experimentally, registering the rate of the ROS production as a measure of the SQ concentration at the Qo site. It is expected that k_c1c_ decreases if the intermembrane space increases, thus diluting the concentration of cytochrome c that is included implicitly in this parameter. The light scattering technique allows measuring changes in the volume of the mitochondrial matrix and, implicitly, the intermembrane space. Light scattering is higher in KCl than in sucrose of the same osmolarity (Fig. 10B). This indicates that the mitochondrial matrix is more compact in KCl than in a sucrose solution. The outer membrane is permeable for both solutes, hence the total mitochondrial volume, which it restricts, must be the same. Therefore the intermembrane space, estimated as the difference between total and matrix volumes, is greater in KCl media. Thus, mitochondria incubated in KCl are characterized by lower values of k_c1c_ than those incubated in sucrose. The experimental results shown in Fig. 10C are consistent with the model. Indeed, in the media with sucrose, the rates of ROS production driven by low succinate concentrations (100–500 uM) are higher than those in KCl supplemented media. In the range of succinate concentrations ≥500 uM, the situation was reversed: ROS production in KCl-based media exceeded that observed in sucrose-based media.

## Discussion

Bifurcation behavior, as revealed by the numerical bifurcation analysis of complex III models, is inherent in the Q-cycle mechanism of electron transport. The main process underlying fold bifurcations in the considered models of complex III was found to be reduction/oxidation and coupled binding/dissociation of ubiquinone in accordance with the Q-cycle mechanism (Text S2). We show here that a decrease of ÄØ accompanied by an increase of ROS production rate can take place as a consequence of perturbations in the respiratory chain operation. The most critical element in such bifurcation behavior is that the same metabolite is reduced at the Qi site and oxidized at the Qo site.

The interaction of complex III with complex I increases the width of the maximal interval of multiple solutions, see Fig. 5, or may even make it infinite, as shown in Fig. 6. The width of this interval is sensitive to the parameter (k_c1c_) that characterizes the combined processes of electron transport from cytochrome c1 to c and further, ultimately reducing molecular oxygen. Thus, it can represent the availability of oxygen and, in this case, the comparison of Figs. 5 and 6, given in Results, demonstrates how hypoxia and reoxygenation may perturb the system to a state of a very high ROS production.

Further extending the model by including into it the reactions of the TCA cycle preserves the interval of parameters where multiple steady state solutions exist. In particular, there are two stable steady states at the parameter values defined in [12] by fitting measured experimental data, as shown in Fig. 7. In experiments performed previously [10], we confirmed that isolated mitochondria incubated with high succinate concentration can persist in one of two different steady states. Mitochondria can be switched from a high ROS production state to a low one by transient incubation with ADP, and then back to a high ROS production state by transient hypoxia. Another experimental confirmation of the predicted behavior of the electron transport chain is the consistency between the predicted curves of steady state levels of SQ at Qo attained at various concentrations of succinate for two different swelling conditions and the measured curves of ROS production rate (Fig. 10).

Stable oscillations that can be obtained at the parameter values in a neighborhood of the Hopf bifurcation point have insignificantly small amplitude, and the region of their stability appears to be so small that it is practically undetectable. However, an increase of the values of the two parameters, which characterize succinate dehydrogenase activity and the rate of combined reactions upstream from cytochrome c_1_, results in the appearance of an interval of succinate concentrations where high-amplitude oscillations exist and are stable (Fig. 9). Feedback interaction of the multistationary respiratory chain operation with the TCA cycle creates oscillations. NADH, as a common metabolite, provides such feedback (see “Mechanism of oscillations” in Result section).

The parameters shown in Tables 1–3 that were used for model 272 were determined from the best fit to the data from experiments performed in vitro in isolated mitochondria. One can expect that the volume of the intermembrane space increased after the procedure of isolation. Such a change of the intermembrane space dilutes cytochrome c, and thus decreases the rate of interaction of cytochromes c_1_ and c. Natural spatial variability of succinate dehydrogenase activity contributes to an uncertainty in its estimated value. Our results show that the change in these parameters, which can be expected in mitochondria of living cells, compared to the isolated ones, results in an oscillatory mode of operation.

In fact, in mitochondria of living cells, flashes and oscillations of ROS production accompanied by the counter-phase changes of ΔΨ can be measured either as a response to laser excitation [13], [14], or as a spontaneous mitochondrial activity [15], [19]–[25]. Usually, the measured *in vivo* decrease of ÄØ that accompanies the ROS flashes was ascribed to either a ROS-induced mitochondrial permeability transition (MPT) [13], [14] or a ROS-activated inner membrane anion channel [26]. Our study opens a new direction in the investigation of the MPT that is of great importance for understanding intracellular signaling and regulation. In particular, it can help to solve the question: why is respiration inhibited during the MPT, when ÄØ is low and cytochrome c still remains in the intermembrane space? Our hypothesis is that the MPT is secondary with respect to the change in the steady state of respiration; it happens when the electron transport chain switches to the “reduced” steady state, where respiration is inhibited by the mechanism considered above. There is more evidence supporting this hypothesis. Matrix pH is well known to be important for the MPT and models considering it as a main factor governing opening/closure of the MPT pore describe the observed events of the MPT [27]. However, the link between the change of matrix pH and the MPT was described phenomenologically; the concrete mechanism of the pH effect on the MPT remains elusive. The models presented here points to the mechanisms by which pH can affect electron transport: protons are explicitly involved in reduction/oxidation of ubiquinone, which is the main process defining the bifurcation. Alkalinization of the matrix must slow down SQ reduction at Qi site and, thus, block electron transport and facilitate the switch to the “reduced” state. If the change to a reduced steady state of the electron transport chain induces the MPT, this provides a concrete mechanism of pH-induced MPT, though it requires further investigation.

Moreover, in some cases, ROS sparks and a decrease of ΔΨ may be a direct consequence of the functional organization of the electron transport, and may not require the involvement of other mechanisms. Thus, many experimentally observed effects, such as excessive ROS production after hypoxia/reoxygenation, or oscillations of ROS production and of ΔΨ, can be explained as a consequence of intrinsic properties of the respiratory chain and its interaction with the central metabolic pathways.

These qualitatively different modes of behavior are manifestations of the same mechanism of electron transport, determined by its quantitative characteristics. Understanding the qualitatively different types of behavior requires a quantitative analysis of electron transport and the linked reactions of the central metabolism. The method presented here can be used for such an objective. However, the simplifications of reality used in our models should be taken into account. In particular, they represent complex III as a monomer, whereas it is known that the functional form is dimeric in living cells [28]. The functional link of monomers at the level of cyt b_L_ was analyzed based on the edge-to-edge distance between cyt b_L_ hemes of the two monomers [29]. It was found that the intermonomer interaction can affect the rate of electron transport, especially in the energized states and when the b_H_ heme is reduced because of a lack of electron acceptors at the Qi site. Using our method to model the dimer would require solving ODE systems containing roughly the square of the number of equations that we analyze here. Such systems can be constructed, but solving them would create computational problems. Performing a preliminary analysis of a simplified model of the bc1 dimer containing cyt b_L_ and b_H_, and Qo binding sites, we found that, despite intermonomer interactions, which quantitatively affect the kinetic behavior of complex III, qualitatively, the dimer demonstrates the same types of bifurcation behavior as the monomer in the situations analyzed in [29].

Another limitation of our models concerns the values for the parameters. The basic set of parameters shown in Tables 1–3 originally was taken from [30] and was then modified by fitting experimental data [12]. In principle, the rate constants can be determined based on the distances of electron tunneling [31], [32]. However, the resolution of 2.1 Å in the determination of distances [33] and uncertainties in other parameters necessary for such determination (as indicated e.g. in [29]) can result in great variation in the values obtained for the rate constants. These uncertainties can be greater than an order of magnitude. The rate constants that we used are inside the range of possible variations admitted by the estimation based on the known distances between the electron carriers.

It should be noted also that the TCA cycle is introduced in the model in a very schematic manner, however, keeping the stoichiometry of respiratory substrates, i.e. succinate and NADH production from pyruvate. Most of the reactions are lumped together and specific reaction mechanisms are not considered. Many of the enzymes performing consecutive reactions form multienzyme complexes [34], [35] (not considered here), where local concentrations of the metabolites can be different from their average concentrations in the matrix. Therefore we did not require average metabolite concentrations at equilibrium to be consistent with respective equilibrium constants. Simulation of a more realistic mechanism of the TCA cycle reactions might affect the bifurcation behavior of the whole system, since, as is indicated above, the appearance and location of Hopf bifurcations and related oscillations is a probable consequence of the interaction of the TCA cycle with reduction/oxidation of ubiquinone. This warrants further investigation.

As is shown by our analysis, perturbations in metabolite concentrations or oxygen availability may induce critical changes in the modes of mitochondrial behavior that result in huge changes in ATP synthesis and ROS production. Changes in the energy supply or signaling or damaging events can have crucial consequences on cell operation in general. We have presented a general overview of the possible modes. At the same time, this approach opens a way to study effects of specific disease conditions on mitochondrial functioning, and to predict mitochondria related disease progressions. In particular, the primary disorder in the chronic obstructive pulmonary disease (COPD) is a decreased capacity of an organism to take up oxygen. The limits of oxygen uptake are measured clinically, and such limitations can be simulated by changing the value of the respective mitochondrial parameter, provided that the other model parameters are determined for the given tissue. In this way the role of the mitochondrial component in a disease progression can be elucidated. A similar approach can be developed, for instance, for diabetes, which results in an essential change in a substrate supply and composition. The effects of such a substrate change on the mitochondrial state and consequences for the whole cell functioning can be predicted. In this way the approach developed here opens a way to better understand progressions of many systemic diseases.

## Methods

### Ethics statement

All procedures involving animals were approved by Children's Hospital of Pittsburgh and were in compliance with “Principles of Laboratory Animal Care” and the current laws of the United States.

### Complex III model

A detailed model of the respiratory chain is described elsewhere [12]. The general algorithm for constructing the ordinary differential equations (ODEs) for the model accounts for all the possible redox states of the respiratory complex III [10] interacting in accordance with the well accepted Q-cycle theory. It assumes that the core of the complex contains four redox sites: cytochrome b with its two hemes, b_H_ and b_L_, cytochrome c_1_, and the iron-sulfur containing Rieske protein (b_H_-b_L_-c_1_-FeS). Each of these redox sites can carry one valence electron. The core can bind the two-electron carrier ubiquinone either on the matrix (Qi) or cytosolic (Qo) side of the inner mitochondrial membrane (b_H_-b_L_-c_1_-FeS-Qo-Qo, Qi-Qi-b_H_-b_L_-c_1_-FeS, Qi-Qi-b_H_-b_L_-c_1_-FeS-Qo-Qo), giving four different configurations of the complex. The model describes binding/dissociation of ubiquinone/ubiquinol that results in interconversion of these four configurations. Bound electron carriers, as well as core redox centers occupy fixed binding sites and have fixed interactions within the respiratory complex. Therefore the probability that a complex is found with a given combination of reduced/oxidized states, including the states of the four redox sites and the states of the substrates, is considered as a variable of the model.

The oxidized state of each redox site is coded as a binary “0” and the reduced state as a binary “1”. In this way various combinations of reduced and oxidized states of carriers can be represented as a four-digit binary numbers with values from 0 to 15 representing redox states of the core (b_H_-b_L_-c_1_-FeS), six-digit binary numbers with values from 0 to 63 representing the redox states of each of two configurations containing one ubiquinone, (Qi-Qi-b_H_-b_L_-c_1_-FeS and b_H_-b_L_-c_1_-FeS-Qo-Qo) and eight-digit binary numbers with values ranging from 0 to 255 representing the redox states of the configurations containing two ubiquinones (Qi-Qi-b_H_-b_L_-c_1_-FeS-Qo-Qo). The algorithm constructs an ODE system for all the configurations and their redox states. This system accounts for the transitions of electrons between carriers resulting in oxidation of the donor (1→0) and reduction of the acceptor (0→1), and binding/dissociation of ubiquinone/ubiquinol. These reactions are simulated in accordance with the well accepted Q-cycle theory and are described in detail in [10].

All the models analyzed here consider the following electron transitions performed by complex III:

from ubiquinol bound at Qo site to the iron-sulfur center of the Rieske protein (qo_FS),
the reverse transition (FS_qo), (eq(6) in [10]),
from the iron-sulfur center of the Rieske protein to cytochrome c1 (FS_c1)
the reverse transition (c1_FS), (eq(7) in [10]),
from the semiquinone bound at the Qo site to the b_L_ heme of cytochrome b (qo_bl)
the reverse transition (bl_qo), (eq(8) in [10]),
from the b_L_ to the b_H_ heme of cytochrome b (bl_bh)
the reverse transition (bh_bl), (eq(9) in [10]),
from the b_H_ heme of cytochrome b to an ubiquinone bound at Qi site (bh_qi1)
the reverse transition (qi1_bh), (eq(9) in [10]),
from the b_H_ heme of cytochrome b to a semiquinone bound at Qi site (bh_qi2)
the reverse transition (qi2_bh) (eq(10 and 11) in [10]),

With one exception (described below) all the models analyzed here consider the following reactions of binding/dissociation of ubiquinone/ubiquinol to/from complex III, (eq(12–16) in [10]),:

- binding of ubiquinol at the Qo site (qhob)
- dissociation of ubiquinol from the Qo site (qhod)
- binding of ubiquinone at the Qi site (qib)
- dissociation of ubiquinone from the Qi site (qid)
- dissociation of ubiquinone from the Qo site (qod)
- binding of ubiquinone at the Qo site (qob)
- dissociation of ubiquinol from the Qi site (qhid)
- binding of ubiquinol at the Qi site (qhib)

The rate constants of the reactions listed above, which were used as a base set of values for the analysis presented in the figures, are shown in Table 1.

### Simplification of complex III model to 257 equations

The numerical continuation algorithm requires that the Jacobian matrix for the model differential equations is nonsingular and its rows are linearly independent. Earlier versions of our model [10], [12] used two binary digits to model the state of ubiquinone, giving 4 combinations (00, 01, 10, and 11), although there are only 3 physically distinct states. In fact, 01 and 10 represent the same state (semiquinone with one valence electron). The algorithm was originally designed so that only the state “01” can be produced, and the amount of configurations containing “10” as a state of semiquinone always was zero. The presence of equations describing such zero-concentration states did not change the result of numerical integration of the initial value problem. However, it did make the Jacobian matrix for the system singular as it contained linearly dependent rows. To perform a bifurcation analysis using CL_MATCONTL, such subsidiary equations had to be eliminated. We modified the algorithm for constructing the ODEs so that it does not include the equations for the states containing “10” semiquinone.

The model simulates electron flow from succinate that reduces ubiquinone. Succinate concentration implicitly defines the maximal rate of ubiquinone reduction:
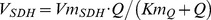
(1)here *Vm_SDH_* = k·S, where S is succinate concentration, k is a constant. Q is ubiquinone concentration. This reaction is accounted for as a term in the differential equation for ubiquinone, which also participates in other electron transport reactions of the respiratory chain. The structurally fixed reduction and oxidation of ubiquinone in complex III respectively on the matrix and cytosolic sides results in the translocation of protons and generation of a transmembrane electric potential.

The conservation of the total amount of complex III and the total amount of ubiquinone is taken into account. The model constructed in this way contains 257 equations (255 equations for the redox states of complex III, and one each for ubiquinone (oxidized), and transmembrane potential).

This and all other models considered here account for a proton leak through the membrane that is exponentially dependent on ΔΨ:
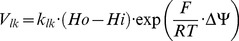
(2)where F = 96500 c/mol is the Faraday constant, R = 8.3 J/(mol×K) is the gas constant, T = 298 K is temperature. Outside (Ho = 0.0001 mM) and matrix (Hi = 0.00005 mM) proton concentrations are considered to be fixed due to high buffer capacity, but ΔΨ is a variable whose dynamics are described by a differential equation that takes into account proton translocations coupled with electron transport [10] and a proton leak described by (2).

### Further simplification of complex III model to 145 equations

In accordance with the Q-cycle mechanism, ubiquinol bound in the Qo site is oxidized giving its electrons to the FeS center of the Rieske protein and cytochrome b_H_ and releasing its protons into the intermembrane space. Then the ubiquinone formed is released. The next pair of electrons can be transported only after the next ubiquinol is bound to the same Qo site. For simplicity, release of ubiquinone and binding of new ubiquinol can be combined and described as an exchange of ubiquinone with ubiquinol at the Qo site. Similarly, the release of ubiquinol and binding of ubiquinone at Qi site can be combined. In this way, all the reactions of the Q-cycle can be described considering only one configuration of complex III that contains two bound ubiquinones (Qi-Qi-b_H_-b_L_-c_1_-FeS-Qo-Qo). After removing “zero-states” containing “10” semiquinone (as described above) and taking into account the conservation of the total contents of complex III and ubiquinone, the above model is reduced to 145 equations.

### Including complex I

The model of 257 equations simulates the whole set of reactions of Q-cycle. The reduction of the number of equations from 400, as explained above, does not change the biological model, but only simplifies its mathematical representation. Therefore we use this simplified set of equations as part of an extended mathematical description of electron transport in the respiratory chain. In addition to the reactions performed in complex III as described above, this extended model with 267 equations accounts for electron transport reactions performed by complex I, as described elsewhere [12]:

reduction of flavin mononucleotide (FMN) oxidizing NADH (fmn) and the reverse reaction (rfmn)
reduction of the center N2 oxidizing FMN (f_n2) and the reverse reaction (n2_f)
reduction of ubiquinone, oxidizing N2, which had previously received the first electron from FMN (n2_q1), and the reverse reaction (q1_n2)
reduction of the semiquinone produced in the previous step, which is bound in the vicinity of N2, taking two protons from the matrix and one electron from ubiquinol bound to the proton well. Oxidation of this latter ubiquinol results in releasing two protons in the intermembrane space (qpqn), and the reverse reaction (rqpqn)
dissociation of the ubiquinol produced in the previous step and simultaneous movement of semiquinone, also produced in the previous step, to the vicinity of N2 (qhdis), and the reverse binding (qhbnd)
reduction of semiquinone oxidizing N2, which had previously received the second electron from FMN (n2_q2), and reverse reaction (q2_n2)
binding of a new ubiquinone (qbnd) and its reverse dissociation (qdis)

The rate constants of the reactions listed above, which were used as a base set of values for the analysis presented in the figures, are shown in Table 2.

Substrate supply in model 267 is treated in the same way as in model 257, with the exception that succinate oxidation depends on NAD^+^. This accounts for NAD^+^-dependent reactions of succinate production in TCA cycle, lumped in the model with SDH and results in NADH production, which is oxidized by complex I:
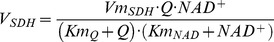
(3)NAD^+^ is a variable of the model and its dynamics are described by a following differential equation that is included in the ODE system describing the dynamics of various redox states of complexes I and III. It accounts for the rate of NAD^+^ production as a result of NADH oxidation by complex I, and the rate of NAD^+^ consumption in the TCA cycle reactions reducing it into NADH:
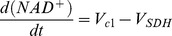
(4)Mass conservation NAD^+^+NADH = const is taken into account (with const = 16 nmol/mg prot).

### Including reactions of the TCA cycle and substrate transport

In this model, consisting of 272 equations, the production of the substrates of respiration, succinate and NADH, is considered in more detail, although still in extremely simplified form. The expressions for some of the reaction rates lump several reactions together and account phenomenologically for the substrates rather than real reaction mechanisms.

Succinate dehydrogenase now accounts for succinate (suc), since it is a real variable of the given model:
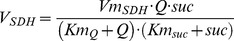
(5)The fumarate oxidation and malate dehydrogenase (MDH) reactions forming oxaloacetate (oa) assume that fumarate and malate are represented as a single pool (mal):
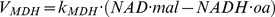
(6)The citrate synthase reaction assumes that pyruvate and acetyl CoA are combined in a single pool (pyr):
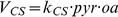
(7)Transport of pyruvate assumes a constant cytosolic concentration (C_pyr_) and a variable mitochondrial concentrations (pyr):
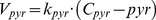
(8)A number of TCA cycle reactions from citrate (cit) to succinate are combined. The whole set depends on citrate as input substrate and NAD^+^:
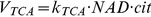
(9)Succinate exchange to fumarate/malate assumes constant external concentrations (C_suc_, C_mal_):
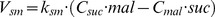
(10)Succinate entry when it is added externally is modeled by:
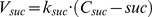
(11)Malic enzyme transforms malate into pyruvate:
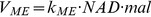
(12)The parameter values for the reactions listed above are shown in Table 3.

Dynamics of the new variables (suc, mal, oa, pyr, cit) are described by following ODEs incorporated in the model:
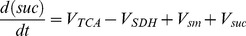
(13)

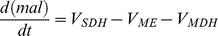
(14)

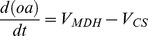
(15)

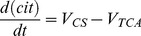
(16)

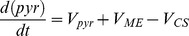
(17)The dynamics of NAD^+^ are now described in a more complex way compared to Eq. (4)

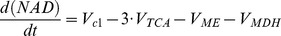
(18)The TCA cycle reactions from citrate to succinate reduce two molecules of NAD^+^ for each succinate produced, but the model also accounts for a molecule of NADH produced by transformation of pyruvate into acetyl CoA, which is not included explicitly.

### Techniques used for the numerical bifurcation analysis

A numerical approach used to systematically search for various types of model behavior was implemented by combining two complimentary techniques: 1) using initial value problem (IVP) solvers to solve IVPs for our ODE systems, and 2) using the numerical bifurcation analysis software CL-MATCONTL to study the corresponding large equilibrium systems.

The first method consists in numerical integration of two IVPs using the same parameter values but different initial states. One initial state is oxidized and one is reduced. Integration is continued until an approximate steady state is reached. A trajectory is considered to have reached an approximate steady state if the time derivatives of all variables were less than 1.0e-12 nmol·(mg prot)^−1^·s^−1^. The numerical solution is obtained using the DASSL method [36], as implemented in the NAG Fortran library (http://www.nag.co.uk/numeric/fl/fldescription.asp). This Fortran code is incorporated within our C++ program. If the steady states reached from the two different initial states are different, this indicates that the given parameters correspond to multiple stable steady state solutions. The algorithm implemented in our software automatically scans parameter regions to find such points of multiple steady states. It detects multiple steady states even if they are located in disconnected branches (as in Fig. 6), but it can detect only stable equilibrium solutions. Parameter values corresponding to multiple steady state solutions are found using this method, and the interval of the chosen parameter is maximized (optionally) as described below. Within the interval thus obtained, we apply the second method to find steady state solution(stable and unstable) and exactly locate and characterize bifurcation points.

The second method consists in numerical continuation and bifurcation analysis of equilibrium solutions to our ODE systems by CL-MATCONTL (Text S1) [17], [18], a MATLAB package for bifurcation analysis of large equilibrium systems. These equilibrium systems are viewed as systems of nonlinear algebraic parameter dependent equations. To compute their solution branches one uses pseudo arch length (numerical) continuation, which is a technique to compute a sequence of consecutive points approximating the desired solution branch using Newton type methods. This allows an accurate computation of both stable and unstable equilibrium solutions on a branch. CL-MATCONTL requires an initial point on the solution branch to start continuation and can compute only a connected solution branch. It can miss a whole branch of steady states, if the complete solution consists of more than one unconnected branches (as in Fig. 6). At each point on the solution branch the Jacobian of the system is computed, and a bifurcation is detected and located when an eigenvalue of the Jacobian crosses the imaginary axis, See Text S1 for more details. Combining both the IVP and continuation methods allows one to find unconnected branches of steady states and various types of steady states and bifurcation points.

### Using method 1 to find the largest region in a parameter space of multiple steady state solutions

#### Step 0. Localization of an interval of multiple solutions

Once a point in parameter space is found corresponding to multiple stable steady state solutions, each approachable from distinct initial values, then an interval of a parameter of interest corresponding to multiple steady state solutions can be determined. In the case of two stable steady states (as shown in Figs. 3 and 4 the method is simple: starting from initial points close to each of the steady states, the algorithm repeatedly increases the parameter of interest and calculates trajectories approaching the steady states, until the steady states that are obtained coincide. The minimal value of the parameter corresponding to a coincidence of solutions from different initial states gives the right boundary of the interval of multiple steady states. The same algorithm applied as the parameter decreases gives the left boundary of the interval.

#### Step 1. Changing a randomly chosen parameter

The width of the interval of the analyzed parameter corresponding to multiple solutions depends on the values of other parameters. The algorithm changes the value of another parameter, randomly chosen from the list of arbitrarily selected parameters to be varied, and repeats Step 0. If the interval of multiple solutions increases after this change, the algorithm accepts the changed value of the parameter, otherwise it rejects the change.

#### Step 2. Passing through the list of parameters

After accepting or rejecting the change, the algorithm randomly chooses another parameter from the list and repeats step 1. This procedure is repeated until changes of all parameters in both directions are checked.

#### Step 3. Maximization of the interval of multiple solutions

Step 2 is repeated many times until no changes after passing through the whole list of parameters are accepted, in other words, when the maximal length of the interval of multiple steady state solutions is reached.

In every calculation the algorithm verifies (by comparing intermediate and last points of solution trajectories) whether a steady state is approached. This verification detects any kind of periodic solutions if they appear in some point.

The C++ source code of our software for automated rule-based construction of large systems of ODEs is available free from http://dl.dropbox.com/u/21877934/plos2012.tar.gz

### Experimental methods

#### Isolation of rat brain mitochondria

All procedures involving animals were approved by Children's Hospital of Pittsburgh and were in compliance with “Principles of Laboratory Animal Care” and the current laws of the United States. Brain mitochondria were isolated from the cortex of adult Wistar rats. After removal, tissue was minced and homogenized in ice-cold isolation buffer I (IB I) which contained: 225 mM mannitol, 75 mM sucrose, 5 mM HEPES buffer (pH adjusted to 7.3 with KOH), 0.1 mg/ml fatty acid free BSA, 1 mM tetrapotassium EDTA and 12% Percoll. The homogenate thus obtained was carefully layered on the top of a discontinuous gradient of Percoll (24% and 42%) prepared using the same buffer. The preparation was then centrifuged at 16,000×*g* for 10 min. The fraction containing the mitochondria located between 42% and 24% Percoll was carefully withdrawn by a syringe and washed from Percoll twice by pelleting in IB I. The resulting mitochondrial suspension was diluted in isolation buffer II (IB II), which was similar to IB I, except for the concentration of EDTA (0.1 mM) and lack of albumin, and spun down at 12,000×*g* for 10 min. The deposit of mitochondria was homogenized in IB II at a final protein concentration of ∼20 mg/ml and stored on ice until use. The protein concentration in the mitochondrial samples was determined using a Protein Assay kit (Pierce Chemical Company, Rockford IL) according to the manufacturer's instructions. Mitochondria prepared in this way were active for at least 5–6 hours, as determined by their ability to maintain a stable transmembrane potential in the presence of oxidizable substrates.

**Fluorescence measurements** were performed in a stirred cuvette mounted in a Shimatzu RF-5301 spectrofluorimeter maintained at 37°C. Mitochondria (0.2 mg/ml of protein) were added to 1.5 ml of the basic incubation medium that contained: 125 mM KCl; 2 mM KH_2_ PO _4_; 2 mM MgCl_2_; 10 mM Tris;10 mM HEPES (pH 7.0); 100 µM EGTA; and oxidizable substrates as indicated in a particular experiment. In sucrose-based media all ingredients were the same, except for KCl which was substituted by 250 mM of sucrose.

**Mitochondrial swelling** was estimated by monitoring light scattering of mitochondrial suspension. It was performed at crossed monochromators with excitation/emission wavelengths 600 nm. Excitation and emission slits were set up for 1.5 nm and 3.0 nm, respectively; the instrument sensitivity was set to the “Low” mode [37].

**Hydrogen peroxide** was measured by fluorescence of Amplex red (2 µM), which increased upon oxidation to resorufin in the presence of 1 U/ml of horseradish peroxidase (HRP) and 20 U/mL of SOD as previously described [37]. Measurements were carried out at excitation/emission wavelengths of 560 nm (slit 1.5 nm)/590 nm (slit 3 nm), respectively. The instrument sensitivity was set to the “High” mode. Amounts of H_2_O_2_ released by mitochondria were estimated by constructing calibration curves using known H_2_O_2_ concentrations in the standard incubation buffer together with Amplex red and HRP, but without mitochondria.

**Mitochondrial respiration rates** were measured by an Oroboros High Resolution Respirometer (Innsbruck, Austria) in a stirred 2 mL chamber at 37°C in the same incubation media as indicated above. The oxygen sensor was calibrated at each experiment according to the manufacturer's instructions. Calculations of respiratory rates were performed by software built into the instrument.

## Supporting Information

Text S1 **Basic principles of Numerical Bifurcation Analysis. CL_MATCONTL and its functionality.** The basics of numerical continuation of equilibrium solutions of ODEs as nonlinear algebraic equations depending on parameters, detecting and locating bifurcations, and also the specific functionality of CL_MATCONTL for Bifurcation Analysis of Large Systems, are described.
(PDF)

Text S2 **Revealing the main process underlying fold bifurcation in model 145.** Asymptotic reduction of the model based on the difference in time scales for model variables allowed to reduce the ODE system to a single equation that contains a fold bifurcation point and points to binding/dissociation of quinones coupled with their reduction/oxidation as a main process defining multi-stationarity of complex III operation.
(PDF)
