# Supplementary material for: Multistationary and Oscillatory Modes of Free Radicals Generation by the Mitochondrial Respiratory Chain Revealed by a Bifurcation Analysis
Source: PLoS Comput Biol. 2012 Sep 20;8(9):e1002700. doi: 10.1371/journal.pcbi.1002700 (PMC3447950; doi:10.1371/journal.pcbi.1002700)
Supplement: Text S1 — Basic principles of Numerical Bifurcation Analysis. CL_MATCONTL and its functionality. The basics of numerical continuation of equilibrium solutions of ODEs as nonlinear algebraic equations depending on parameters, detecting and locating bifurcations, and also the specific functionality of CL_MATCONTL for Bifurcation Analysis of Large Systems, are described. (PDF) [file pcbi.1002700.s001.pdf]

Basic principles of Numerical Bifurcation Analysis. CL_MATCONTL and its functionality

Large systems of equilibrium solutions of the ODEs of interest are systems of nonlinear algebraic equations depending on parameters. They are studied by the methods of numerical bifurcation analysis, which is based on continuation. The principal approach of numerical bifurcation analysis is based on continuation of solutions to well-defined operator equations. Such computational results give a deeper understanding of the solution behavior, stability, multiplicity, and bifurcations, and often provide direct links to underlying mathematical theories.

We first summarize the basic principles of *Numerical Bifurcation Analysis.*

Numerical Continuation
Consider an autonomous differential equation

$$du/dt = f(u,\alpha), \text{ where } u \in R^n,\ \alpha \in R, f(u,\alpha) \in R^n. \qquad (1)$$

Suppose we want to compute a one-parameter family of equilibria of (1), that is, a *solution branch* of

$$f(u,\alpha) = 0 \qquad (2)$$

To compute these solution branches one uses *numerical continuation*, which is a technique to compute a sequence of consecutive points approximating the desired branch. The continuation algorithm uses a predictor-corrector method that requires solving nonlinear systems by a Newton type method at each continuation step.

Detecting and Locating Bifurcations
Let $x(s) = (u(s),\alpha(s)) \in R^n \times R$ be a smooth local parameterization of a solution branch of the system (2). We write the Jacobian matrix along this path as $A(s) := f_u(x(s))$. A *bifurcation point* on a solution branch is a point where at least one eigenvalue of the Jacobian matrix $A(s)$ has zero real part. A *test function* $\phi(s) := \psi(x(s))$ is a (typically) smooth scalar function that has a regular zero at a bifurcation point. A bifurcation point between consecutive continuation points $x(s_k)$ and $x(s_{k+1})$ is *detected* when

$$\psi(x(s_k))\psi(x(s_{k+1})) < 0. \qquad (3)$$

The bifurcation point is *located typically* by applying Newton's method to an extended system

$$\begin{cases} f(x) = 0, \\ g(x) = 0, \end{cases} \qquad (4)$$

CL-MATCONT *and its extension* CL-MATCONTL.
CL-MATCONT and its GUI version MATCONT (Dhooge et al., 2003) are MATLAB packages for the study of dynamical systems and their bifurcations for small and moderate size problems.

Recently, Friedman and his collaborators developed the Continuation of Invariant Subspaces (CIS) algorithm for computing a smooth orthonormal basis for an invariant subspace R(s) of a parameter-dependent matrix $A(s) \in R^{n\times n}$, $s \in [0,1]$. [1,2]. The CIS algorithm uses projection methods to deal with large problems. In applications, R(s) is a low-dimensional invariant subspace of $A(s)$ responsible for bifurcations.

The CIS algorithm was incorporated into Cl_matcont to extend its functionality to large scale bifurcation computations via subspace reduction as follows.

Standard bifurcation analysis algorithms, such as those used in CL-MATCONT, involve computing functions of $A(s)$. These methods were adapted Cl_matcontL to large problems by computing the same functions of a much smaller restriction $C(s) := A(s)|_{R(s)}$ of $A(s)$ onto $R(s)$. Note that the CIS algorithm ensures that only eigenvalues of $C(s)$ can cross the imaginary axis, so that $C(s)$ provides all the relevant information about bifurcations. In addition, the continued subspace is adapted to track behavior relevant to bifurcations.

The resulting code is Cl_matcontL [3 - 9].

Basic functionality of CL_MATCONTL for Bifurcation Analysis of Large Systems

Computation and continuation of bifurcations of equilibria (1) in the system (2).

1) Detecting and locating codimension-1 bifurcations on the equilibrium curve:
   a) fold (or limit point)
   b) Hopf
2) Locating a simple branch point on the equilibrium curve and switching branches.
3) Continuation of codimension-1 bifurcations:
   a) Fold
   b) Hopf
4) Locating codimension-2 bifurcations on a curve of codimension-1 bifurcations:
   a) Locating codimension-2 points on the fold curve: Bogdanov-Takens (or double zero) bifurcation, Fold-Hopf bifurcation
   b) Locating codimension-2 points on the Hopf curve.

1. J. Demmel, L. Dieci, and M. Friedman, "Computing connecting orbits via an improved algorithm for continuing invariant subspaces", *SIAM J. Sci. Comp.*, **22**, No. 1 (2001), 81 - 94.
2. L. Dieci and M. Friedman, "Continuation of Invariant Subspaces", *Numer. Lin. Alg. Appl.* **8** (2001), 317 - 327.
3. D. Bindel, J. Demmel, M. Friedman, W. Govaerts, and Yu.A. Kuznetsov "Bifurcation analysis of large equilibrium systems in Matlab". In: V.S. Sunderam et al. (eds.) "Proceedings of the International Conference on Computational Science ICCS 2005, Atlanta, GA, USA, May 22-25, 2005, Part I". Springer Verlag *Lecture Notes in Computer Science* 3514 (2005), 50-57.
4. M. Friedman, W. Govaerts, and Yu.A. Kuznetsov, and B. Sautois, "Continuation of Homoclinic Orbits in Matlab" In: V.S. Sunderam et al. (eds.) "Proceedings of the International Conference on Computational Science ICCS 2005, Atlanta, GA, USA, May 22-25, 2005, Part I". Springer Verlag *Lecture Notes in Computer Science* 3514 (2005), 263-270.
5. M. Friedman and W. Qiu, "On the location and continuation of Hopf bifurcations in large-scale problems", *Int. J. Bifur. & Chaos*. **18**, No 5 (2008), 1589-1597.
6. D. Bindel, J. Demmel, M. Friedman, "Continuation of Invariant Subspaces in Large Scale Bifurcation Problems", *SIAM J. Sci. Comp.* **30**, No 2 (March 2008), 637-656.
7. J. Hughes and M. Friedman, "A bisection-like algorithm for branch switching at a simple branch point", J. Sci. Comput. **41**, No 2 (2009), 62-69.
8. D. Bindel, M. Friedman, W. Govaerts, J. Hughes, Yu.A. Kuznetsov, "Cl_matcontL2.2, *Continuation Toolbox in MATLAB*" University of Alabama in Huntsville, January 2010, http://webpages.uah.edu/~hughesjs/.
9. D. Bindel, M. Friedman, W. Govaerts, J. Hughes, Yu.A. Kuznetsov, "CL_MATCONTL2.2 Tutorial", University of Alabama in Huntsville, January 2010, http://webpages.uah.edu/~hughesjs/.
