# Supplementary material for: Multistationary and Oscillatory Modes of Free Radicals Generation by the Mitochondrial Respiratory Chain Revealed by a Bifurcation Analysis
Source: PLoS Comput Biol. 2012 Sep 20;8(9):e1002700. doi: 10.1371/journal.pcbi.1002700 (PMC3447950; doi:10.1371/journal.pcbi.1002700)
Supplement: Text S2 — Revealing the main process underlying fold bifurcation in model 145. Asymptotic reduction of the model based on the difference in time scales for model variables allowed to reduce the ODE system to a single equation that contains a fold bifurcation point and points to binding/dissociation of quinones coupled with their reduction/oxidation as a main process defining multi-stationarity of complex III operation. (PDF) [file pcbi.1002700.s002.pdf]

Revealing the main process underlying fold bifurcation in model 145.

In the general model, there are three types of equations describing three types of processes that take place in the simplest model 145 describing a single configuration of complex III (Qi-Qi- b_H – b_L -FeS- c_1 -Qo-Qo, see main text). These hree types of processes have different characteristic times:

1. equations describing electron transport between the redox states (Ci, Cj), with the rate constants k_{ij} and $k_{ji,}$ that do not require binding/dissociation. They represent the main part of the reactions of the model. In a general form, they can be written as:

$$\frac{dC_i}{dt} = -\sum k_{ij} \cdot C_i + \sum k_{ji} \cdot C_j \qquad (1)$$

2. equations describing transitions between the configurations by binding/dissociation of quinones:

$$\frac{dx_0}{dt} = k_i \cdot Qx \cdot Q - k_o \cdot x_0 \cdot QH_2 \qquad (2a)$$

$$\frac{dxQ}{dt} = k_o \cdot x_0 \cdot QH_2 + k_i \cdot QxQ \cdot Q - 2 \cdot k_e \cdot xQ \qquad (2b)$$

$$\frac{dQx}{dt} = k_e \cdot xQ - k_i \cdot Qx \cdot Q - k_o \cdot Qx \cdot QH_2 \qquad (2c)$$

$$x_0 + xQ + Qx + QxQ = c_0 \qquad (2d)$$

3. reduction/oxidation of quinones:

$$\frac{dQ}{dt} = k_o \cdot x_0 \cdot QH_2 + k_o \cdot Qx \cdot QH_2 - k_i \cdot Qx \cdot Q - k_i \cdot QxQ \cdot Q - v_{SDH} \qquad (3a)$$

$$QH_2 + Q = q_0 \qquad (3b)$$

Here k_i is the rate constant of the combined reaction of QH_2 dissociation and Q binding at the Qi site; k_o is the rate constant of the combined reaction of Q dissociation and QH_2 binding at the Qo site; k_e is the rate constant of electron transport reactions from Qo to Qi; x_0 is the sum of forms with both the Qo and the Qi sites oxidized; Qx is the sum of the forms with the Qi sites reduced and Qo oxidized; xQ is the sum of the forms with the Qi sites oxidized and the Qo reduced; QxQ is the sum of the forms with both Qo and Qi sites reduced. The factor of 2 in equation 2b takes into account that two molecules of QH2 must be oxidized at the Qo site in order to reduce one Q at the Qi site.

Electron transport (1) proceeds much faster than binding/dissociation and can be considered to be in a quasi steady state. Each variable of the system (2) in fact represents the sum of a number of various redox states of the complex combined in accordance with the redox state of the Qi and Qo sites that determine the possibility of binding/dissociation. Thus, assuming the apparent difference in time scales between the electron transport and binding/dissociation, we consider the reduced systems (2) and (3), which implicitly include steady state solution of equations (1).

This reduced system was further analysed using Tikhonov's asymptotic reduction [1]. To facilitate the analysis we transform the system to unitless variables. Dividing eq (2a) by total concentrations c_0 and q_0 allows to transform to unitless concentrations $z_0=x_0/c_0$, $qz=Qx/c_0$, $qh=QH_2/c_0$, $q=Q/c_0$:

$$\frac{dz_0}{q_0 \cdot dt} = k_i \cdot qz \cdot q - k_o \cdot z_0 \cdot qh$$

assuming unitless time $\tau = t \cdot c_0 \cdot k_i$, gives:

$$\frac{c_0 \cdot dz_0}{q_0 \cdot d\tau} = qz \cdot q - k'_o \cdot z_0 \cdot qh \qquad (4a)$$

where $k'_o = k_o / k_i$

Similar transformation of equations 2b-2d gives

$$\frac{c_0 \cdot dzq}{q_0 \cdot d\tau} = k'_o \cdot z_0 \cdot qh + qzq \cdot q - 2 \cdot k'_e \cdot zq \qquad (4b)$$

$$\frac{c_0 \cdot dqz}{q_0 \cdot d\tau} = k'_e \cdot zq - qz \cdot q - k'_o \cdot qz \cdot qh \qquad (4c)$$

$$z_0 + zq + qz + qzq = 1 \qquad (4d)$$

Equations (3) are transformed into

$$\frac{dq}{d\tau} = k'_o \cdot z_0 \cdot qh + k'_o \cdot qz \cdot qh - qz \cdot q - qzq \cdot q - v'_{SDH} \qquad (5a)$$

$$qh + q = 1 \qquad (5b)$$

Here qzq and zq are unitless transformations of QxQ and xQ, $k'_e = k_e / k_i / q_0$, $v'_{SDH} = v_{SDH} / k_i / q_0$.

After the transition to unitless concentrations and time, for the sake of simplicity of description, we preserve the notation k_e, k_o, and v_{SDH} for the unitless quantities (since they have the same meanings as the ones initially presented in equations (2) and (3).

The concentration q_0 is about an order of magnitude higher than the one c_0. Thus, strictly speaking, c0/q0 cannot be considered as a small parameter, however it indicates that the relative changes of the forms of the complex III are faster and it sooner reaches a quasi-equilibrium than the ubiquinone does.

In a quasi-equilibrium, equations (4) take the form:

$$qz \cdot q - k'_o \cdot z_0 \cdot qh = 0 \qquad (6a)$$

$$k'_o \cdot z_0 \cdot qh + qzq \cdot q - 2 \cdot k'_e \cdot zq - k'_o \cdot qz \cdot qh = 0 \qquad (6b)$$

$$k'_e \cdot zq - qz \cdot q = 0 \qquad (6c)$$

$$z_0 + zq + qz + qzq = 1 \qquad (6d)$$

Solving the equations (6) for z_0, qz, zq, and qzq, gives:

$$z_0 = k_e \cdot q^2 / den\,;\ qz = qh^2 \cdot q \cdot k_o^2 + qh \cdot q^2 \cdot k_o / den\,;\ zq = qh \cdot k_e \cdot q \cdot k_o / den\,;\ qzq = 2 \cdot qh^2 \cdot k_e \cdot k_o^2 - qh \cdot k_e \cdot q \cdot k_o / den\,;$$

here

$$den = 2 \cdot qh^2 \cdot k_e \cdot k_o^2 + 2 \cdot qh \cdot k_e \cdot q \cdot k_o + qh^2 \cdot q \cdot k_o^2 + k_e \cdot q^2 + qh \cdot q^2 \cdot k_o$$

Substituting this solution into (5a) results in

$$\frac{dq}{d\tau} = num / den - v_{SDH} \qquad (7)$$

where

$$num = k_o \cdot k_e \cdot q^2 \cdot qh + k_o \cdot qh^2 \cdot q \cdot k_o^2 + qh \cdot q^2 \cdot k_o \cdot qh - qh^2 \cdot q \cdot k_o^2 + qh \cdot q^2 \cdot k_o \cdot q - 2 \cdot qh^2 \cdot k_e \cdot k_o^2 - qh \cdot k_e \cdot q \cdot k_o \cdot q$$

Thus, eq. (7) represents a simplification of the whole system to a single equation.

The steady state solution of the system reduced to one equation (7) corresponds to the derivative equal to zero:

$$num / den - v_{SDH} = 0 \qquad (8)$$

The graph of the steady state equation (8) (taking into account eq (5b)) in two variables: concentration of the free ubiquinone q and v_{SDH} that represents the succinate concentration, is given in Figure 1. This curve has a typical shape of a fold bifurcation. Thus, the analysis indicates that the oxidation/reduction of the ubiquinone, coupled with binding/dissociation at the Qo and Qi sites, can be considered as the main process determining fold bifurcation.

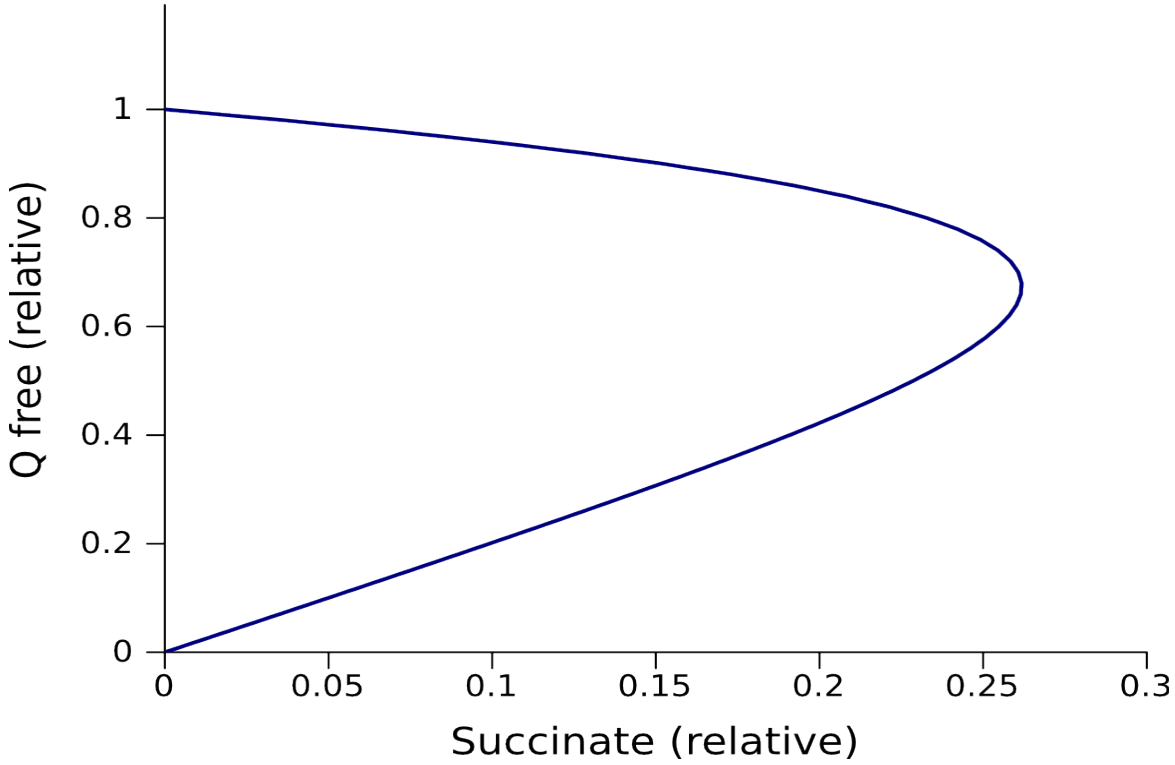

Figure S1. Steady state concentration of free ubiquinone as a function of succinate concentration represented by v_{SDH}. Other parameters: k_o=2, k_e=90.

References

1. Tikhonov AN. (1952) Systems of differential equations, containing small parameters at the derivatives. Mat. Sbornik 31: 575–586.
